# Self-Limited Formation of Bowl-Shaped Nanopores for Directional DNA Translocation

**DOI:** 10.1021/acsnano.1c06321

**Published:** 2021-11-11

**Authors:** Ngan Hoang Pham, Yao Yao, Chenyu Wen, Shiyu Li, Shuangshuang Zeng, Tomas Nyberg, Tuan Thien Tran, Daniel Primetzhofer, Zhen Zhang, Shi-Li Zhang

**Affiliations:** † Division of Solid-State Electronics, Department of Electrical Engineering, Uppsala University, SE-751 03 Uppsala, Sweden; ‡ Division of Applied Nuclear Physics, Department of Physics and Astronomy, Uppsala University, SE-751 20 Uppsala, Sweden

**Keywords:** solid-state nanopores, self-limiting formation, directional DNA translocation, silicon technology, local oxidation of silicon (LOCOS), bowl shape, electroosmotic effects

## Abstract

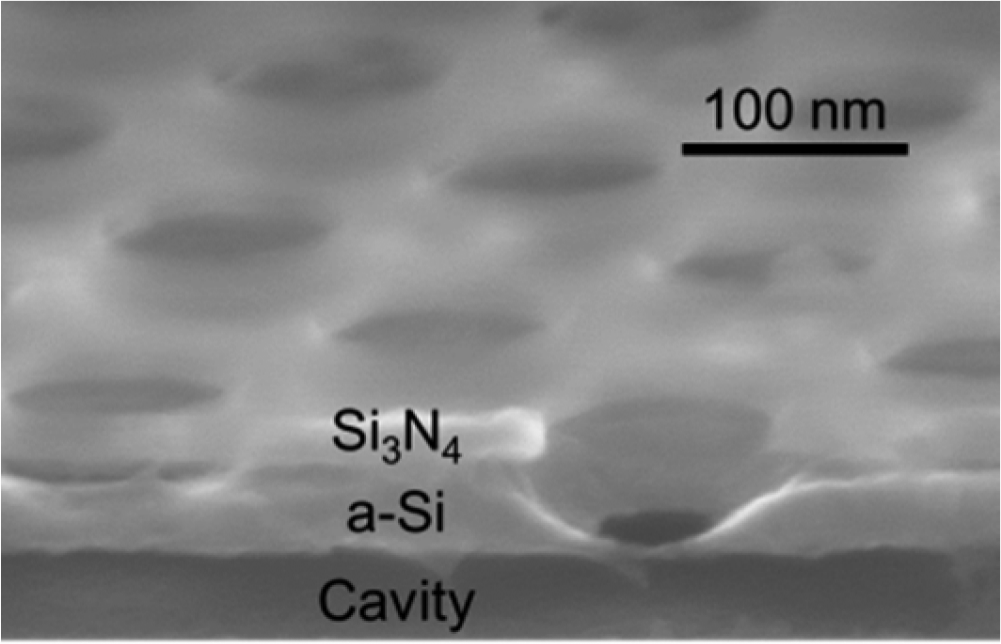

Solid-state nanopores of on-demand dimensions and shape can facilitate desired sensor functions. However, reproducible fabrication of arrayed nanopores of predefined dimensions remains challenging despite numerous techniques explored. Here, bowl-shaped nanopores combining properties of ultrathin membrane and tapering geometry are manufactured using a self-limiting process developed on the basis of standard silicon technology. The upper opening of the bowl-nanopores is 60–120 nm in diameter, and the bottom orifice reaches sub-5 nm. Current–voltage characteristics of the fabricated bowl-nanopores display insignificant rectification indicating weak ionic selectivity, in accordance to numerical simulations showing minor differences in electric field and ionic velocity upon the reversal of bias voltages. Simulations reveal, concomitantly, high-momentum electroosmotic flow downward along the concave nanopore sidewall. Collisions between the left and right tributaries over the bottom orifice drive the electroosmotic flow both up into the nanopore and down out of the nanopore through the orifice. The resultant asymmetry in electrophoretic–electroosmotic force is considered the cause responsible for the experimentally observed strong directionality in λ-DNA translocation with larger amplitude, longer duration, and higher frequencies for the downward movements from the upper opening than the upward ones from the orifice. Thus, the resourceful silicon nanofabrication technology is shown to enable nanopore designs toward enriching sensor applications.

Nanopore technology has been intensively investigated for a myriad of applications including biological analysis,^1^ water desalination,^2,3^ gas separation,^4,5^ selective filtering,^6,7^ and power generation.^8,9^ Solid-state nanopores (SSNPs) have attracted particular interest because they can be manufactured using a variety of fabrication techniques along with a rich choice of membrane materials both mechanically and chemically robust.^10^ The techniques employed include drilling using ions^11,12^ or electron beam,^13^ electrical breakdown,^14^ electrochemical etching,^15^ and lithography in combination with reactive ion etching.^16,17^ Tightly connected is the processable membrane materials including SiN_*x*_,^11^ SiO_2_,^18^ and silicon^19^ that are silicon process compatible; emerging layered single-atom structures such as graphene^20^ and MoS_2_^21^ that can facilitate ultrathin nanopores; or glass^22^ and polymer^23,24^ that target special routes of fabrication and surface management. However, the techniques for SSNP fabrication reported in the literature often require tedious optimization and precise time control in order to achieve target pore dimensions. A self-limiting process with which the dimensions can be preset and do not vary upon, *e.g*., prolonged process time, is desired for reproducible fabrication. The present work exploits the resourceful silicon nanofabrication technology for manufacturing SSNPs in a self-limiting and mass-production fashion. The specific process module employed to attain the self-limiting formation is the local oxidation of silicon (LOCOS) established in standard silicon technology for the electrical isolation of devices in integrated circuits before transistors reached submicron dimensions.^25^ A natural consequence of the LOCOS process is the formation of bowl-shape SSNPs, hereon abbreviated as BNPs. It is worth noting that this module is applicable for SSNP formation on any low-noise substrates able to withstand high-temperature processing. The quasi-hemispheric interior with an ultrathin membrane around the orifice of BNPs is experimentally and theoretically found to give rise to weak ionic selectivity for the transport of ions but strong molecular directionality for translocations of DNA molecules.

## Results and Discussion

### Fabrication of the BNPs

**Figure 1:**
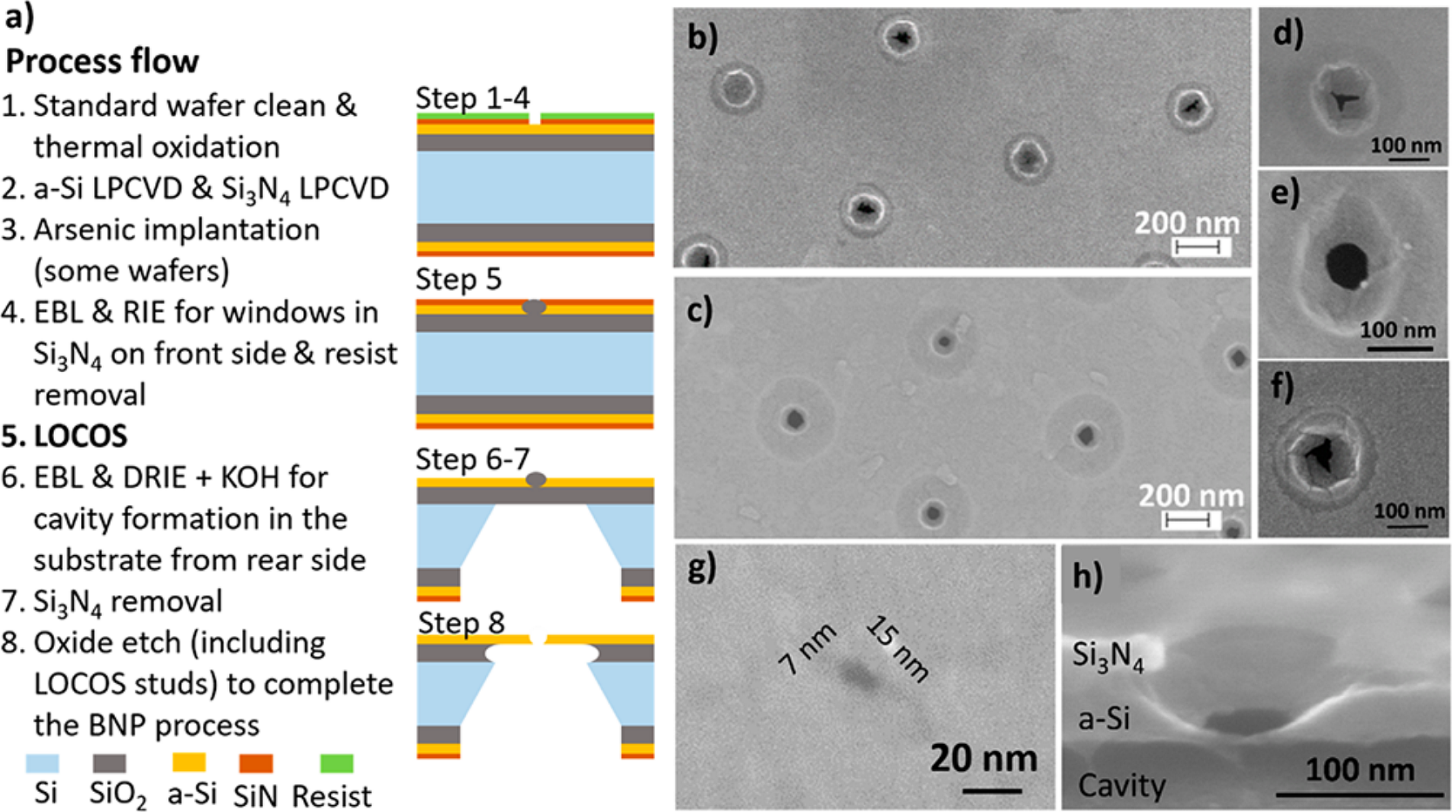
BNP fabrication. (a) Overview assisted by graphical presentation of key process steps for the formation of BNPs. Top-view SEM micrographs showing two BNP arrays (b) with their irregular bottom orifices in a nonimplanted sample after the LOCOS process at 850 °C for 150 min and (c) with much more regular bottom orifices in an implanted and activated sample after the LOCOS process at 1100 °C for 60 min. Close-in of the bottom orifice for: (d) one of the BNPs in (b); (e) one of the BNPs in (c); and (f) a different BNP formed after oxidation at 850 °C for 150 min of the crystallized a-Si layer implanted with As. (g) Top-view of a BNP with an elliptic bottom orifice in a sample similarly processed as that in (e) but with a smaller opening in the Si_3_N_4_ layer. (h) Tilted cross-sectional view of a similar BNP as that in (e).

The BNPs were fabricated on (100) silicon wafers thinned from 550 to 325 μm in thickness and double-side polished. Graphically summarized in Figure 1a, the fabrication started from thermal oxidation of the wafers at 1000 °C to grow a 150 nm thick SiO_2_ layer after standard wafer cleaning. Low-pressure chemical vapor deposition (LPCVD) was used to grow a 60 nm amorphous silicon (a-Si) membrane layer at 560 °C. Oxidation at 850 °C for 10 min was performed to grow a screening SiO_2_, thereby slightly reducing the a-Si layer to 55 nm, followed by the growth of 20–70 nm thick Si_3_N_4_ at 775 °C. The a-Si layer was inevitably crystallized during the oxidation and the Si_3_N_4_ deposition, but it is still referred to as a-Si for convenience despite its polycrystalline form. Ion implantation of arsenic to a dose of 5 × 10^15^ cm^–2^ was carried out to the front side of some of the wafers at 20 keV so as to place the dopant at the interface between the a-Si layer and the Si_3_N_4_/SiO_2_ overlayer. These wafers were annealed in an inert atmosphere at 850 °C for 60 min to electrically activate the dopants. The Si_3_N_4_ layer that received the arsenic implantation was stripped off in H_3_PO_4_ solution at 170 °C after the high-activation annealing. A fresh LPCVD-Si_3_N_4_ layer 20 nm in thickness was deposited on these wafers to ensure a quality LOCOS process together with the wafers without the arsenic doping. Electron beam lithography (EBL, Nanobeam Ltd.) was combined with reactive ion etching (RIE) to define circular windows of 60 nm in diameter in the Si_3_N_4_ layer on the front side of the wafers. During LOCOS (Step 5 in Figure 1a), the a-Si exposed in the Si_3_N_4_ windows was oxidized in dry oxygen at atmospheric pressure from 850 to 1100 °C for different time spans in order to evaluate the BNP process. With a minor surface oxidization, the oxidation-resistant Si_3_N_4_ layer remained largely intact.^25^ The BNP fabrication was completed by combining deep RIE and KOH wet etch to form square cavities 150 μm × 150 μm in size from the rear side through the silicon substrate until reaching the 150 nm thick SiO_2_ layer. The preferential chemical reaction of KOH with the (100) planes to the (111) planes of a silicon crystal defined the sloped sidewalls at a fixed angle of the large cavities.^17^ Finally, a buffered hydrofluoric acid was applied to remove the LOCOS SiO_2_ studs grown in the Si_3_N_4_ windows along with the SiO_2_ layer in the large cavities. Self-supporting silicon membranes with BNPs were obtained. The fabricated BNPs were inspected by means of scanning electron microscopy (SEM).

The BNPs formed with critically different process specifics are first reviewed. This is followed by evaluation of the BNPs based on numerical simulations and experimental demonstrations with transport of ions and translocation of λ-DNA strands. Technical details regarding the simulation and electrical characterization implementations are provided in the Methods section and the Supporting Information to keep the discussion below focused.

### Structure Characterization and Formation Optimization

Use of the highly stiff LPCVD Si_3_N_4_ layer with a Young’s modulus of ∼440 GPa^26^ is essential to the self-limiting formation of BNPs *via* the LOCOS process. The top-view micrographs in Figure 1b,c present typical BNP arrays formed with LOCOS (b) at 850 °C for 150 min of the crystallized a-Si membrane and LOCOS (c) at 1100 °C for 60 min of the crystallized a-Si first implanted and subsequently activated with arsenic. The bottom orifices of the BNPs in Figure 1b are highly irregular, in contrast to those in Figure 1c displaying, as desired, largely circular orifices that well-resemble the shape of the top opening of much larger dimensions. Close-in micrographs of Figure 1b,c are, respectively, depicted in Figure 1d,e (orifice diameter *d*_p_ = 65 nm). To highlight the importance of arsenic activation, the close-in micrograph of a BNP processed similarly to those in Figure 1c,e but without the activation step shows persistent irregularity in Figure 1f. The top-view micrograph in Figure 1g displays a BNP with an elliptic orifice (*d*_p1_ = 7 nm and *d*_p2_ = 15 nm) resembling fairly well the shape of the top opening of much larger dimensions. The micrograph in Figure 1h depicts the cross-section with a 15° tilt of a BNP formed similarly to that in Figure 1e to show a concavely curved sidewall.

Several causes can be identified as responsible for the observed nonuniformities and irregularities. To begin with, the windows in the Si_3_N_4_ layer can be nonuniform during lithography: (a) distortion from the desired circular shape and (b) size variation from window to window. Process optimization is necessary to minimize these lithography related nonuniformities. The irregularities in orifice size and shape can result from two major variabilities: (a) dependence of oxidation rate on the silicon crystal orientation^27^ of the crystallized a-Si and (b) local thickness variation clearly inferred by the roughened surface in Figure 1h. The relatively satisfying result with a close-to-ideal circular orifice in Figure 1e points to the paramount importance of doping prior to LOCOS, because heavily doped silicon is known to oxidize faster and the rate of oxidation is less dependent on crystal orientation than undoped silicon.^28^ Prolonging the oxidation time while exploiting the self-limiting nature of the process may also improve the uniformity. Alternatively, such irregularities could be diminished by substantially increasing the grain size in the a-Si layer *via* grain growth mediated by metal or metal silicides.^29,30^ The local thickness variation is most likely a consequence of crystallization and grain growth in the a-Si layer upon thermal processing. Chemical mechanical polishing, a standard process technique in modern nanoelectronics fabrication, can be employed to planarize the surface prior to further processing. Finally, the membrane is extremely thin in the nanometer range in the vicinity of the BNP orifice. Such BNPs can be mechanically fragile. To boost the mechanical strength, the BNPs can be coated by an ultrathin dielectric layer in a conformal manner by means of atomic layer deposition.^31^ Thickened orifices can compromise the performance of the BNP sensors, but simulations indicate that the adverse effect is limited.

**Figure 2:**
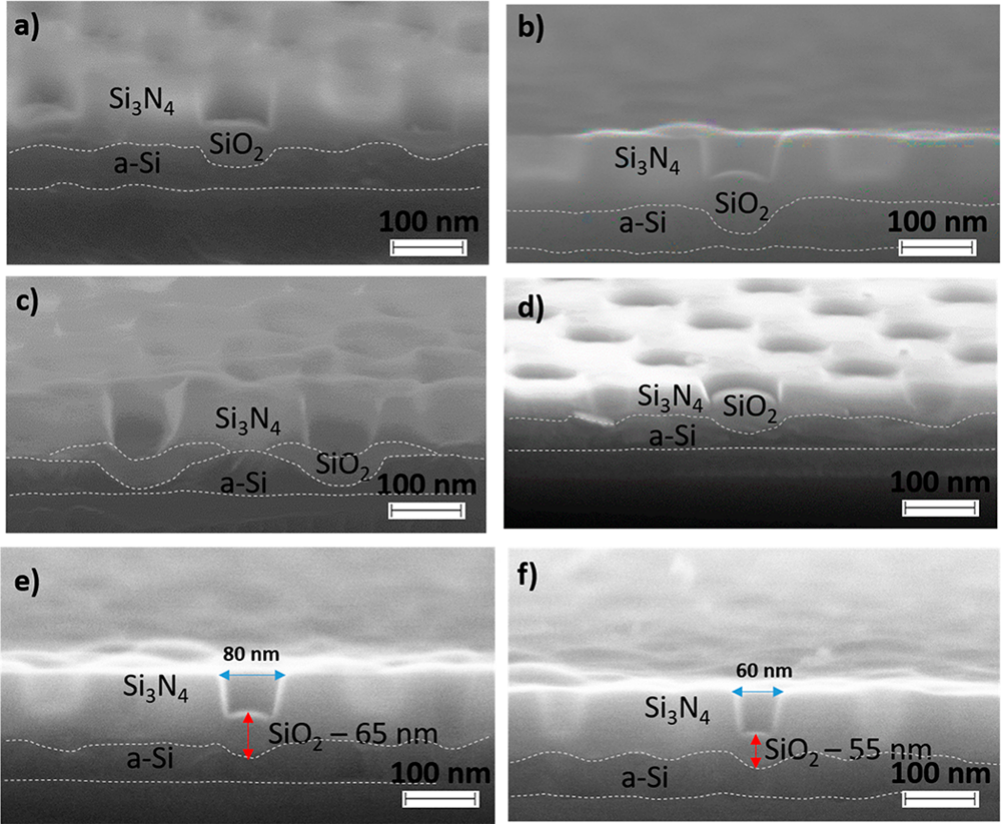
Self-limiting formation of BNPs. Cross-sectional SEM images of BNP arrays showing self-limiting effects. Incomplete oxidation by the mechanical stress imposed by a 70 nm thick, high-stiffness Si_3_N_4_ layer after LOCOS (a) at 850 °C for 60 min; (b) at 850 °C for 180 min; and (c) reoxidation at 850 °C for 60 min of the sample in (b) after stripping off the SiO_2_ stud that formed during the first oxidation. In comparison, (d) sample with a 40 nm thick Si_3_N_4_ layer after LOCOS at 850 °C for 150 min. Size-dependent growth of SiO_2_ studs at 850 °C for 180 min: (e) a thicker SiO_2_ stud in an 80 nm opening than (f) that in a 60 nm opening. Note that the interface between the a-Si layer and the underlying SiO_2_ should be flat, and the wavy white dashed lines are due to artifacts from manual cleavage of the samples.

The mechanical stress generated during the LOCOS process^25^ can be engineered to result in a self-limiting growth of SiO_2_ studs as the nanopore precursors defined by the Si_3_N_4_ windows. When the SiO_2_ studs are selectively stripped off, nanopores are left behind in the a-Si membrane. Here, the stress considered specifically refers to that in the grown SiO_2_ studs. The oxidation of silicon is accompanied by a 220% volume expansion.^25^ Confined in a stiff environment, 120% of a grown SiO_2_ stud has to stick out through the Si_3_N_4_ window, thereby rendering the growth of SiO_2_ stud self-constricting. Moreover, the diffusivity of oxidants is greatly reduced^32^ in a highly compressed SiO_2_, thus shifting the silicon oxidation toward self-restricting. Stress engineering can be viable in several different approaches including varying the Si_3_N_4_ thickness, altering the Si:N ratio from 3:4 in Si_3_N_4_ to 1:0.8 to yield lower-stiffness SiN_0.8_,^26^ and/or controlling the LOCOS temperature. Such approaches aim at allowing the grown SiO_2_ stud to push back the upper SiN_*x*_ layer so as to yield room for further growth. The cross-sectional SEM images in Figure 2 clearly show the outcome of the subtle, yet critical, interplay between stress management *via* Si_3_N_4_ thickness control and LOCOS SiO_2_ growth. With the assistance of white dashed lines to identify the interfaces of the different parts in the structure, incomplete oxidation of the a-Si inside a 100 nm diameter window in a 70 nm thick Si_3_N_4_ layer is seen in Figure 2a, with a ∼ 30 nm gap between the front of the grown SiO_2_ stud and the bottom of the a-Si/SiO_2_ interface. The self-limiting phenomenon is evident in Figure 2b as the SiO_2_ stud had only thickened by 30% upon a 3-fold longer oxidation of the sample in Figure 2a; the grown SiO_2_ on a planar silicon substrate would be 110 nm thick after the 60 min oxidation and 250 nm upon prolonging the oxidation time to 180 min.^25^ An effective route to work around the stress as an attempt to advance the SiO_2_ stud toward the bottom of the a-Si/SiO_2_ interface is to first strip it off followed by reoxidization of the sample, as shown in Figure 2c. Decreasing the thickness of the Si_3_N_4_ layer from 70 to 40 nm reduces the required LOCOS time for the same oxide thickness, as revealed in Figure 2d. Further decrease of the Si_3_N_4_ layer to 20 nm is proven an effective scheme to lessen the mechanical constriction imposed on the growing SiO_2_ stud so as to lead to a successful BNP process, cf., Figure 1e,h. The stress buildup is naturally dependent on window size, *i.e*., Figure 2e *vs.* 2f. Stress engineering along with the design of ion implantation with dose, energy, and incidence angle can additionally be utilized to determine the concavity of the BNP interior, which requires process optimization supported by simulation. Thus, the self-limiting growth of SiO_2_ and the subsequent selective oxide removal should be key to the formation of highly reproducible BNPs.

The self-limiting growth of SiO_2_ due to mechanical stress needs a warranted supply of oxidant (dry O_2_ in this study) to the reaction interface between the partially grown SiO_2_ stud and the remaining a-Si. The kinetics of SiO_2_ thermal growth on single-crystal silicon has been intensively studied and well-described by the widely accepted Deal–Grove model.^33^ The underlying mechanism is the diffusion of oxidants through the growing SiO_2_ and the oxidation reaction with the Si at the Si–SiO_2_ interface to thicken the oxide. Therefore, the SiO_2_ growth rate in steady-state is determined by^33^ (a) the flux of oxidants from the gas bulk to the SiO_2_ surface, *F*, (b) the solid solubility of oxidants in the oxide, (c) the diffusivity of oxidants in the oxide, and (d) the interface reaction coefficient. The original Deal–Grove model is one-dimensional, valid for single-crystal silicon, and without consideration of doping effects. Comprehensive studies on the dependence of oxidation rate on silicon crystal orientation^27^ and dopant type and concentrations^28^ are readily available in the literature. For oxidation over severe surface morphology with sharp concave or convex corners, a two-dimensional model^32,34^ can be used to account for the stark mechanical stress and oxidant supply effects. The supply and consumption of oxidants are estimated for our case as follows.

On the supply side, *F* in m^–2^s^–1^, of oxidant molecules from the gas toward a solid surface with a given partial pressure, *p* in mTorr, is^35^, with *k* = 1.38 × 10^–23^ J K^–1^ as the Boltzmann constant, *m* as the molecular mass in kg, and *T* as the absolute temperature in K. In our case of oxidation in pure O_2_ (*m*_O_2__ = 5.3 × 10^–26^ kg) at *p* = 760 Torr (atmospheric pressure) and *T* = 1123 K, *F* = 1.4 × 10^27^ m^–2^ s^–1^ was obtained. The opened windows for the BNP formation are typically 110 nm in diameter, giving rise to each of the exposed window areas to be 9.5 × 10^–15^ m^2^. Thus, there are 1.3 × 10^13^ O_2_ molecules striking the considered area per second.

On the consumption side, the oxidation rate can be related to the flux of oxidant at the Si/SiO_2_ interface, *F*_I_, according to the relationship^33^, where *x*_0_ is the thickness of grown SiO_2_, *t* is the oxidation time, and *N*_1_ is the number of oxidant molecules incorporated per unit volume of SiO_2_ grown. For our dry oxidation in O_2_, *N*_1_ = 2.2 × 10^22^ cm^–3^, *x*_0_ = 120 nm, and *t* = 180 min. The area of the Si/SiO_2_ interface is approximated by a hemisphere of radius = 55 nm (a-Si membrane thickness) at the end of BNP formation. Therefore, the amount of oxygen consumed would be 5.6 × 10^3^ O_2_ molecules per second at the Si/SiO_2_ interface, *i.e*., 10^9^ times lower than the supply. This strongly suggests that there should be no shortage in the oxidant supply for oxidation in such small openings and that the observed self-limiting oxidation is caused by mechanical stress. Refinements of this simplified calculation can consider several details, some of which have already been discussed above. The presence of *n*-type dopants can enhance the oxidation rate of (100) oriented silicon by a factor of 10 at the most,^28,36^ while the crystal orientation can change the oxidation rate by 1.7 times.^27^ The SiO_2_ growth in the small Si_3_N_4_ windows is three-dimensional with the oxidation of a polycrystalline, heavily arsenic doped silicon membrane layer. Hence, the nonuniform structures can introduce several mechanisms to be considered:^32,34,37−39^ (a) effects of different crystal orientations of a curved silicon surface on the local oxidation rate, (b) dependence of oxidant diffusion on the shape of the SiO_2_/Si boundary surfaces reflecting the level of mechanical strain, (c) viscous flow in the nonuniformly deformed SiO_2_, and (d) effects of stress on oxide growth rate. Nevertheless, none of these three-dimensional effects are expected to cause orders-of-magnitude changes to revert the conclusion of mechanical stress being the cause for self-limiting oxidation.

The importance of arsenic activation in achieving the desired circular orifice deserves some lines of discussion. By invoking a correlation of the oxidation rate constant to the Fermi level of silicon, a theoretical model was proposed to account for the enhancement effect by heavy arsenic doping.^40^ The oxidation of heavily arsenic doped polycrystalline silicon is more complex, as the dopants can redistribute and deactivate.^25^ In polycrystalline silicon, the segregation of *n*-type dopants to the grain boundaries of a polycrystalline silicon film is a prominent phenomenon leading to severe dopant deactivation.^41^ At an elevated temperature, an initially amorphous silicon layer recrystallizes and simultaneously incorporates preimplanted arsenic atoms to the substitutional lattice sites. This incorporation process has an activation energy of 2.3 eV.^42^ For comparison, the silicon oxidation is characterized by an activation energy of 2.05 eV.^43,44^ Hence, the oxidation of the a-Si without prior arsenic activation (Figure 1f) is expected to proceed similarly to that of the nonimplanted a-Si (Figure 1d).

### Electroosmotic Flow Distribution and Ionic Selectivity

**Figure 3:**
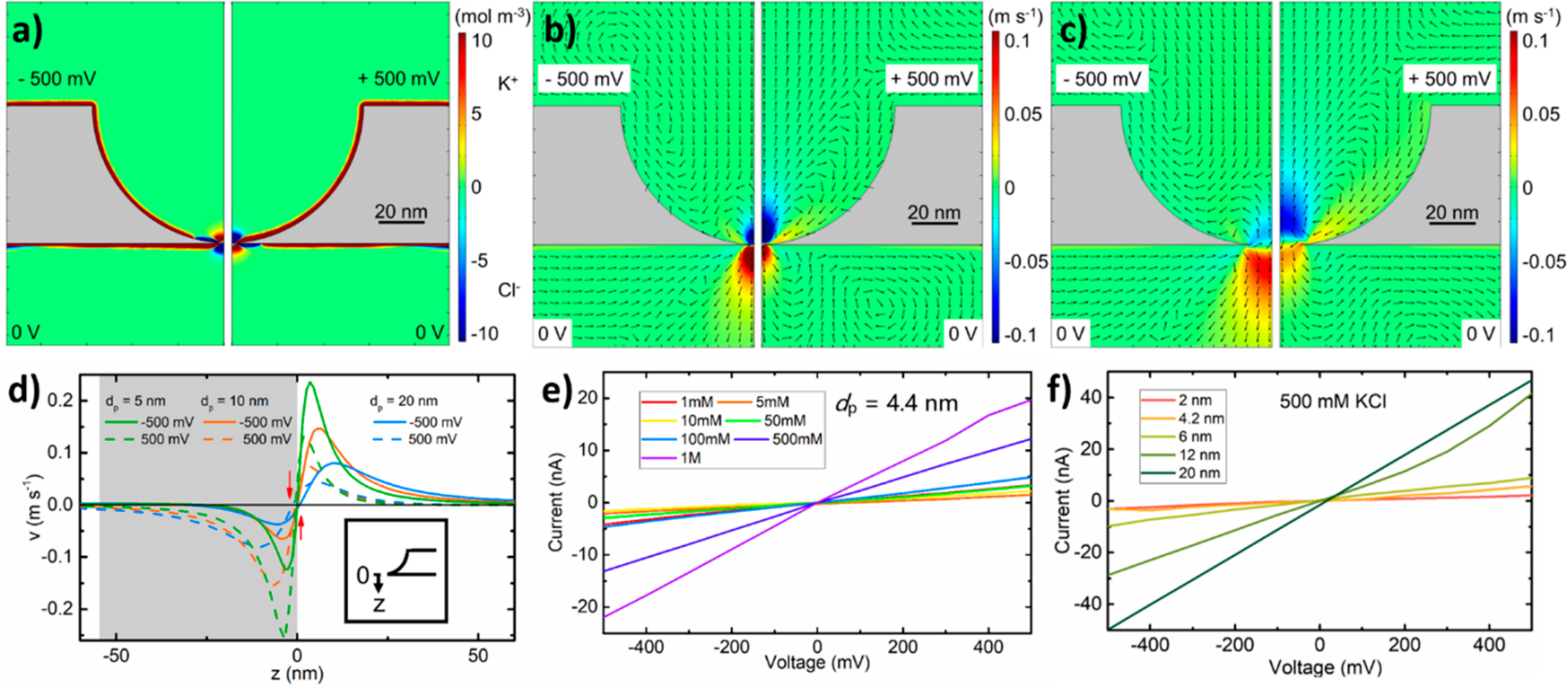
Characterization of BNPs by the means of simulation and experiment. (a) Distribution of net charge concentration in a BNP of *d*_p_ = 5 nm (warm colors for cations and cold colors for anions), clearly showing an extended stretch of inverted charge due to the induced charge effect for both bias polarities, but above (−500 mV) and below (+500 mV) the membrane around the orifice. Electroosmotic flow velocity distribution (warm colors for downward flows and cold colors for upward flows) at −500 mV (left half) and +500 mV (right half) for (b) *d*_p_ = 5 nm and (c) *d*_p_ = 20 nm. The direction of each of the arrows is assigned on the basis of the data from the center point of the arrow. (d) Electroosmotic flow velocity along the symmetrical axis for *d*_p_ = 5, 10, and 20 nm. Current–voltage characteristics of (e) a fabricated BNP of *d*_p_ = 4.4 nm in electrolytes of various KCl concentrations and (f) fabricated BNPs of *d*_p_ = 2–20 nm in an electrolyte of KCl at a concentration of 500 mM.

The BNPs were first studied by means of two-dimensional numerical simulation with a central axial symmetry implemented on Comsol Multiphysics, with details about the procedure described in the Methods section as well as in our recent review.^10^ Referring to the experimental BNPs, extended ideal hemispheres with a radius of 55 nm, equal to the a-Si membrane thickness split to two halves at the bottom, and distanced by *d*_p_ = 5–20 nm, equal to the orifice of BNPs, were adopted in the simulation. The simulation also assumed the frequently measured σ = −0.02 C m^−2^ for the inherent surface charge density, in 500 mM KCl and biased at ±500 mV. It is clear in Figure 3a for a BNP of *d*_p_ = 5 nm that induced charge effect^45^ gives rise to a nonuniform charge distribution along the curved sidewall of the BNP and, in particular, a charge inversion on the upper surface at −500 mV and the lower surface at +500 mV of the ultrathin membrane around the orifice. This charge nonuniformity has a direct effect on the electroosmotic flow (EOF) in Figure 3b for a *d*_p_ = 5 nm BNP and Figure 3c for a *d*_p_ = 20 nm BNP. Use of the same color scale makes it easy for a direct comparison between Figure 3b and 3c. As the sidewall is negatively charged until the vicinity of the orifice, the EOF originating from the drift of cations (K^+^ ions) in the electrical double layer glides downward along the hemispherical sidewall at +500 mV, in analogy to water flowing on a curved slide. This EOF is entirely dominated by its horizontal component at the end (bottom) of the hemispherical slide. By virtue of axial symmetry, fluid splash occurs over the plane of the bottom orifice (orifice plane) as a result of head-to-head collisions between each pair of the electroosmotic tributaries of equal momentum from the opposite side across the orifice. The splash causes the fluid to flow both upward back into the BNP and downward outside the orifice, seen, respectively, as cold-color and warm-color “flames” in the right halves of Figure 3b,c. As a result, a sharp and flat border between the flames lies close to the horizontal line defined by the tangent of the hemisphere at the orifice, *i.e*., the orifice plane. The splash together with the EOF along the two surfaces generates three electroosmotic vortexes in the vicinity of the orifice, one inside the BNP and two in the lower electrolyte reservoir. The two vortexes close to the orifice are characteristic of the bowl shape, while the vortex more distanced from the orifice can also form in ordinary symmetric, *e.g*., cylindrical, nanopores.^46^ Moreover, the vortex right outside the orifice results from the charge inversion on the lower surface close to the orifice (blue stretch in the right half of Figure 3a). The peculiar distribution of EOF in the BNP results from two kinds of surface charge, inherent and induced, present along the nanopore sidewall. The latter arises from the parasitic capacitive effect of the membrane. The negative inherent charge is, *via* attraction of positive ions in the electrical double layer, responsible for the water flow gliding along the sidewall from the high to the low-potential side of the BNP. The induced surface charge thrusts, in a similar fashion, two streams of flows along the upper and lower surface of the membrane toward the orifice where they collide and move forward.^47^ The final apparent EOF distribution is the superposition of the patterns caused by the two kinds of surface charge. Largely similar results are found when the BNPs are biased negatively at −500 mV but with the relative size of the flames reversed, as shown in the left halves of Figure 3b,c. The reversion in flame size and intensity but not in color (flow direction) is a consequence of two collaborative effects. One is the induced charge of opposite polarity on the upper surface of the ultrathin membrane around the orifice (blue stretch in the left half of Figure 3a), causing a local downward flow (left halves of Figure 3b,c) and driving a similar fluid splash over the orifice plane. Another is the fast-moving EOF mostly horizontally along the lower surface leading to two fluid splash components, one by the parallel collision with the downward flow from the upper surface of opposite charge polarity^48^ and one, again, by the head-to-head collision between opposite tributaries. The effect of fluid splash is illustrated by plotting the EOF velocity along the central axis for the BNPs with *d*_p_ = 5, 10, and 20 nm in Figure 3d with the inset for the coordinate. The position of the sharp transition from one velocity polarity to the opposite shifts slightly with increasing *d*_p_, as indicated by the vertical red arrows. The shift, accompanied with the change of flame size and intensity, is expected because the electroosmosis arising from the charge on the sidewall is weaker for larger pores, *i.e*., Figure 3b *vs*. 3c, and it is a response to the bias condition.

Well-characterizing current–voltage (*I–V*) curves of the BNPs are shown in Figure 3e,f. The ionic rectification, defined as the ratio averaged from 0 to ±500 mV of ionic current at a specific negative bias voltage to that at the counterpart positive voltage, is insignificant in the studied ranges of KCl concentration (Figure 3e) or *d*_p_ (Figure 3f and Figure S1). The membrane near the orifice of the BNPs is extremely thin and it is located in the high-electric field region that determines the electrical behavior in the open-pore state. Thus, the distribution of the electric field in the BNPs is similar to that in conventional symmetrical two-dimensional nanopores,^20,21^ and so are the leaner *I–V* characteristics (Figure S2). In addition, the open-pore current shows a typical noise power spectrum with a normal noise level comparable to that of SiN_*x*_ nanopores (Figure S3).^49^ The *d*_p_ values were extracted on the basis of the *I–V* data and our model (Note S1 and Figure S4 for details).^50^ In addition, the surface charge density is extracted to be around −0.017 C m^−2^ using the conductance data of the nanopore in electrolytes of different KCl concentrations (Figure S5).

### Force Components and λ-DNA Translocation

**Figure 4:**
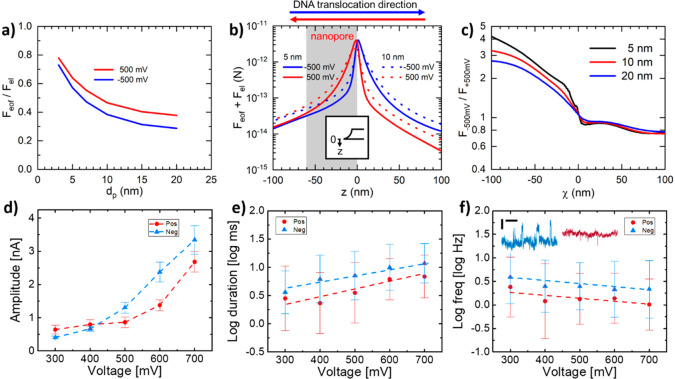
Characterization of BNPs by the means of simulation and experiment. (a–c) COMSOL simulations for BNPs of *d*_p_ = 5–20 nm, *h* = 55 nm (thickness of the membrane) and *D*_p_ = 115–130 nm (diameter of the upper opening that is equal to the diameter of the hemisphere plus *d*_p_), in 500 mM KCl and with σ = −0.02 C m^−2^ inherent surface charge density. (a) Dependence on *d*_p_ of the maximum ratio of the electroosmotic force to the electrophoretic force at the same position. (b) Total force on the DNA molecule along the central axis of BNPs of *d*_p_ = 5 and 10 nm. Inset: the coordinate with the origin lining up with the orifice plane and pointing to the lower reservoir. (c) Variation for BNPs of *d*_p_ = 5, 10, and 20 nm of the corresponding ratio of the total force at negative bias to that at positive bias along the χ-axis, defined as the direction of DNA translocation, *i.e*., from the upper reservoir to the lower reservoir at negative bias (−500 mV) and from the lower reservoir to the upper reservoir at positive bias (+500 mV). (d–f) Translocation of DNA in an 8 nm BNP. Dependence on bias of: (d) average amplitude of translocation spikes, (e) average translocation duration, and (f) mean FTE. Dots represent average values, while error bars define the spread of corresponding quantities. Dash lines are a view guide for a better illustration. Red, positive bias; blue, negative bias. Inset in (f): Typical current trace segments showing translocation spikes at −500 mV (blue) and +500 mV (dark red). The horizontal and vertical scale bars are 0.5 s and 0.2 nA, respectively.

In our previous report of truncated-pyramidal nanopores, the observed strong rectification of protein translocation was associated with the formation of electroosmotic vortex.^17^ As protein molecules are generally weakly charged, the electroosmotic vortex plays a critical role in the dynamics of their translocation while the electrophoretic force is relatively unimportant. In contrast, ionization of the hydrogen on their phosphate backbone makes DNA molecules heavily negatively charged.^51^ Movement of DNA molecules in a BNP can, therefore, be controlled electrophoretically and significantly modulated electroosmotically.^10,52^ At +500 mV (Figure 3b), the electrophoretic force would pull a DNA molecule upward from the lower reservoir. However, the electroosmotic force below the orifice tends to drag the molecule downward while that above pushes it upward. Thus, the electroosmotic force would attempt to prevent the DNA molecule from entering the BNP from the lower reservoir but would drive it out from the BNP (once it is inside) into the upper reservoir. At −500 mV, the direction of the electrophoretic force is reversed but the EOFs near the pore only change in intensity. A DNA molecule driven by the electrophoretic force to enter the BNP from the upper reservoir would become decelerated by the upward EOF when it gets close to the orifice. A long transfer time for the DNA molecule in the BNP is, therefore, anticipated. Similarly, the DNA molecule is accelerated once it moves out into the lower reservoir. The maximum amplitude ratio of the electroosmotic force to the electrophoretic force at the same position along the central axis is calculated in Figure 4a for a simplified assessment using one DNA base pair segment as a nanoscale object being exerted by the different forces (Note S2 and Figures S6 and S7 for calculation). A rapid growth of the importance of the electroosmotic force with decreasing *d*_p_ is evident. The total force, *i.e*., the sum of electrophoretic and electroosmotic force, exerted on such a DNA segment is shown in Figure 4b to peak sharply near the orifice. In both cases of *d*_p_ = 5 and 10 nm, the peaks are strongly asymmetric although the peak heights are largely insensitive to *d*_p_. Further, a comparison between the BNPs and truncated conical nanopores^17^ shows striking differences in how the amplitude ratio evolves with bias voltage (Figure S8).

The ratio of the total force at −500 mV to that at +500 mV on the DNA segment is depicted in Figure 4c for three BNPs of *d*_p_ = 5, 10, and 20 nm. The χ-axis, with the position of the orifice plane as its origin (inset in Figure 4b), points to the direction of the electrophoretic force for both positive and negative biases. The total force at −500 mV is about 5 times stronger than that at +500 mV for DNA approaching the orifice of the *d*_p_ = 5 nm BNP, whereas it is about 30% stronger at +500 mV than that at −500 mV after it passes through the orifice. Hence, the DNA segment is much more strongly attracted to the orifice from the upper reservoir at negative bias, but it is more easily pulled out from the BNP to the upper reservoir at positive bias. Longer duration and higher frequency for DNA translocation are, then, predicted at negative bias than at positive bias. It is also found in Figure 4c that the ratio decreases with increasing *d*_p_ in the χ < 0 domain and that changing *d*_p_ causes little difference in this ratio for driving the DNA segment out from the BNP in the χ > 0 domain.

The λ-DNA translocation experiments were then implemented on a BNP of *d*_p_ = 8 nm. The pore size was similarly extracted from the conductance data. The amplitude, duration, and frequency of translocation events (FTE) were extracted from the original records of ionic current traces. The average and spread of these three parameters are compared for different biases in Figure 4d–f (scatter plots in Figure S9 showing the amplitude and duration distribution of translocation events). The amplitude (Figure 4d) increases significantly with the absolute bias voltage, consistent with other reports.^20,31,53,54^ The amplitude at negative biases is generally higher than that at positive biases. The difference in λ-DNA translocation in the opposite directions of the BNP results in asymmetric ion concentration and electric field distribution across the nanopore, which in their turn leads to the temporal DNA-translocation induced amplitude asymmetry at different bias polarities. Because of the high diffusion coefficient of ions in the electrolyte, the ion distribution tends to rapidly recover to that of the open-pore state after translocation, thereby showing a symmetric electric field distribution as well as nearly linear *I–V* curves. This is special for the BNPs. Although their dependency on bias voltage is weak, both duration (Figure 4e) and FTE (Figure 4f) are consistently higher at negative biases than at positive biases. An effective capture radius^55^ is defined as that of the equipotential surface at potential *D*/μ, with *D* and μ being the diffusivity and mobility of DNA, respectively. The potential on the capture surface for dsDNA is estimated to be 5.4 × 10^–4^ V (Note S3 and Figure S10 for details). From the simulation data, the capture radius is read around 1.4 μm at negative bias and 1.2 μm at positive bias. These numbers indicate a higher capture rate and translocation frequency at negative bias, in good agreement with the experimental data. The observed weak bias-dependent FTE is a result of the competition between capture probability and temporary clogging,^31^ compounded by the rapid increase in the ratio of electroosmotic force to electrophoretic force. The inset of Figure 4f with typical examples of current traces at −500 mV (blue) and +500 mV (red) visualizes the noteworthy differences in amplitude, duration, and FTE. The difference in FTE at positive and negative biases is more significant in smaller BNPs of *d*_p_ = 4.4 and 6 nm; the observed translocation waveforms for the BNP of *d*_p_ = 4.4 nm imply a strong interaction between the BNP and the translocating DNA (Figure S11). Apart from the asymmetric electroosmotic force, other factors including the DNA configuration within the pore especially for strongly confining nanopores^56^ can also contribute to the voltage-direction-dependent DNA translocation.

Fluctuations in current are contributed by translocation spikes, baseline tremble, drift, and background noise. Thus, the translocation of λ-DNA at negative biases is more likely to take place and the interaction between the λ-DNA and the BNPs is stronger, since the former leads to more spikes while the latter contributes to an unstable baseline. The outstanding DNA translocation phenomena are closely correlated to the special structure of and the EOF distribution in the BNPs displayed in Figure 3a–d. Hence, it is experimentally observed and theoretically supported that the DNA molecules have a higher probability to translocate at negative biases than at positive biases in the BNPs.

## Conclusion

A self-limiting process based on standard silicon technology has been developed for the formation of BNPs with attractive properties characteristic of ultrathin membrane around the orifice and the special electroosmotic patterns due to the presence of surface charge on the bowl-shaped sidewall. The former that could be helpful in boosting sensing resolution that shows weak ionic selectivity, while the latter leads to a strong directionality of DNA translocation with several times of difference in amplitude, duration, and frequency for DNA translocation.

## Methods

### COMSOL Simulation

Numerical simulations based on COMSOL Multiphysics of bowl-shaped and truncated conical nanopores with similar dimensions of the experimental BNPs were carried out to gain insights into the nanopore physics. The simulation included the fluid domain and the membrane domain whose relative permittivity was set to 80 and 11.7 for water and silicon, respectively. The ionic movement in an electrolyte was governed by the Nernst–Planck equation, while the electric potential distribution was described by the Poisson equation and the fluid flow was determined by the Navier–Stokes equations. The “Transport of Diluted Species” module (Nernst–Planck equation), the “Electrostatics” module (Poisson equation), and the “Laminar Flow” module (Navier–Stokes equations) were incorporated and fully coupled in our two-dimensional axisymmetric simulation. The mobilities of K^+^ and Cl^–^ were 7.0 × 10^–8^ and 7.2 × 10^–8^ m^2^ V^–1^ s^–1^, respectively. The respective diffusion coefficient was then determined through the Einstein relation. With a rotational symmetry along the central axis of the nanopore, the distributions of electric field and electroosmotic flow were plotted in cross-section along this axis.

### DNA Sample Preparation

λ-DNA was purchased from Merck KGaA, Darmstadt, Germany. The DNA was used without further purification and dispersed in a 500 mM KCl solution to a concentration of 78 pM (2.5 μg mL^–1^).

### Electrical Characterization and Data Processing

In all measurements, the bias voltage was set to the upper reservoir on the large opening side of the BNP, while the lower reservoir on the smallest restriction side was grounded, in agreement with the configuration in the COMSOL simulations.

Before measurement, the BNP chips were thoroughly cleaned in a piranha solution with H_2_SO_4_/H_2_O = 3:1 (volume ratio) at 80 °C for 30 min and rinsed in deionized water. The chips were then sandwiched by two custom-made poly(methyl methacrylate) lids with two polydimethylsiloxane O-rings (inner diameter 8 mm) on the two sides for seal. An inlet and an outlet were made in each lid, and the KCl solution with a certain concentration and λ-DNA dispersions could be injected to the two sides of the nanopore chip. The fluids in the two sides were separated by the chip, and the only path of ionic current was through the nanopore. The resistivity of the KCl solution was calibrated using a conductivity meter (Lab 945, Xylem Analytics Germany Sales GmbH & Co. KG). A pair of pseudo Ag/AgCl reference electrodes (2 mm in diameter (Warner Instruments LLC.)) was also mounted in the middle of the lids, which was used to apply a bias voltage and to measure the ionic current. The bias voltage was controlled and the ionic current was recorded using a patch clamp amplifier (Axopatch 200B, Molecular Device Inc.). The ionic current was digitalized by an Axon Digidata 1550A (Molecular Device LLC.) and recorded using the software Axon pCLAMP 10 (Molecular Device LLC.). DNA translocation was detected at a 10 kHz sampling frequency with a 1 kHz four-pole Bessel low-pass filter. The entire experimental setup was placed inside a Faraday cage to shield against electromagnetic interference.

The translocation spikes on the current traces were extracted automatically by employing a MATLAB program using the function *findpeaks*. The threshold for recognition of a possible spike in the program was set to be eight times the root-mean-squared value of the background noise. The amplitude and duration of each translocation spike were extracted, and the translocation frequency was calculated as the reciprocal of the time interval between consecutive events. In addition, the general fluctuation of the current traces was evaluated by their standard deviation. The components in a high-frequency range (1 kHz–10 Hz) and a low-frequency range (<10 Hz) were separated using a filter, and the standard deviation was calculated separately.

## Supporting Information Available

Figures and notes of ionic rectification ratio of fabricated BNPs with different diameters, electric field distribution in a 5 nm diameter BNP, noise power spectrum density of BNP, calculation of *L*_eff_ of the BNPs, *I–V* characteristics of a 4.4 nm BNP and conductance–conductivity curve, distribution of the total force acting on a base pair of dsDNA, distribution of the electrophoretic force and the electroosmotic force along the central axis of the BNP, electroosmotic flow velocity distribution, comparison between BNP and TCP (truncated conical nanopore) for dependence on bias voltage, scatter plots of amplitude of translocation spikes of λ-DNA translocating an 8 nm BNP, electrical potential distribution in an 8 nm diameter BNP, and translocation traces and typical waveform of λ-DNA translocating the 4.4 nm BNP and discussions of resistance model for BNP pore size and surface charge extraction, dominance of electrophoretic force during dsDNA translocation, and estimation of capture radius of nanopore for DNA translocation (PDF)

## References

[ref1] Miles B. N.; Ivanov A. P.; Wilson K. A.; Doğan F.; Japrung D.; Edel J. B. Single Molecule Sensing with Solid-State Nanopores: Novel Materials, Methods, and Applications. Chem. Soc. Rev. 2013, 42, 15–28. 10.1039/C2CS35286A. 22990878

[ref2] O’Hern S. C.; Jang D.; Bose S.; Idrobo J.-C.; Song Y.; Laoui T.; Kong J.; Karnik R. Nanofiltration across Defect-Sealed Nanoporous Monolayer Graphene. Nano Lett. 2015, 15, 3254–3260. 10.1021/acs.nanolett.5b00456. 25915708

[ref3] Amadei C. A.; Montessori A.; Kadow J. P.; Succi S.; Vecitis C. D. Role of Oxygen Functionalities in Graphene Oxide Architectural Laminate Subnanometer Spacing and Water Transport. Environ. Sci. Technol. 2017, 51, 4280–4288. 10.1021/acs.est.6b05711. 28333448

[ref4] Dong G.; Hou J.; Wang J.; Zhang Y.; Chen V.; Liu J. Enhanced CO_2_/N_2_ Separation by Porous Reduced Graphene Oxide/Pebax Mixed Matrix Membranes. J. Membr. Sci. 2016, 520, 860–868. 10.1016/j.memsci.2016.08.059.

[ref5] Park H. B.; Kamcev J.; Robeson L. M.; Elimelech M.; Freeman B. D. Maximizing the Right Stuff: The Trade-Off between Membrane Permeability and Selectivity. Science 2017, 356, eaab0530 10.1126/science.aab0530. 28619885

[ref6] Walker M. I.; Ubych K.; Saraswat V.; Chalklen E. A.; Braeuninger-Weimer P.; Caneva S.; Weatherup R. S.; Hofmann S.; Keyser U. F. Extrinsic Cation Selectivity of 2D Membranes. ACS Nano 2017, 11, 1340–1346. 10.1021/acsnano.6b06034. 28157333 PMC5333182

[ref7] Caglar M.; Silkina I.; Brown B. T.; Thorneywork A. L.; Burton O. J.; Babenko V.; Gilbert S. M.; Zettl A.; Hofmann S.; Keyser U. F. Tunable Anion-Selective Transport through Monolayer Graphene and Hexagonal Boron Nitride. ACS Nano 2020, 14, 2729–2738. 10.1021/acsnano.9b08168. 31891480 PMC7098055

[ref8] Feng J.; Graf M.; Liu K.; Ovchinnikov D.; Dumcenco D.; Heiranian M.; Nandigana V.; Aluru N. R.; Kis A.; Radenovic A. Single-Layer MoS_2_ Nanopores as Nanopower Generators. Nature 2016, 536, 197–200. 10.1038/nature18593. 27409806

[ref9] Zhang Y.; He Y.; Tsutsui M.; Miao X. S.; Taniguchi M. Short Channel Effects on Electrokinetic Energy Conversion in Solid-State Nanopores. Sci. Rep. 2017, 7, 46661 10.1038/srep46661. 28440281 PMC5404231

[ref10] Wen C.; Zhang S.-L. Fundamentals and Potentials of Solid-State Nanopores: A Review. J. Phys. D: Appl. Phys. 2021, 54, 023001 10.1088/1361-6463/ababce.

[ref11] Li J.; Gershow M.; Stein D.; Brandin E.; Golovchenko J. A. DNA Molecules and Configurations in a Solid-State Nanopore Microscope. Nat. Mater. 2003, 2, 611–615. 10.1038/nmat965. 12942073

[ref12] Li J.; Stein D.; McMullan C.; Branton D.; Aziz M. J.; Golovchenko J. A. Ion-Beam Sculpting at Nanometre Length Scales. Nature 2001, 412, 166–169. 10.1038/35084037. 11449268

[ref13] Storm A. J.; Chen J. H.; Ling X. S.; Zandbergen H. W.; Dekker C. Fabrication of Solid-State Nanopores with Single-Nanometre Precision. Nat. Mater. 2003, 2, 537–540. 10.1038/nmat941. 12858166

[ref14] Goto Y.; Yanagi I.; Matsui K.; Yokoi T.; Takeda K. Integrated Solid-State Nanopore Platform for Nanopore Fabrication *via* Dielectric Breakdown, DNA-Speed Deceleration and Noise Reduction. Sci. Rep. 2016, 6, 31324 10.1038/srep31324. 27499264 PMC4976334

[ref15] Feng J.; Liu K.; Graf M.; Lihter M.; Bulushev R. D.; Dumcenco D.; Alexander D. T. L.; Krasnozhon D.; Vuletic T.; Kis A.; Radenovic A. Electrochemical Reaction in Single Layer MoS_2_: Nanopores Opened Atom by Atom. Nano Lett. 2015, 15, 3431–3438. 10.1021/acs.nanolett.5b00768. 25928894

[ref16] Ahmadi A. G.; Peng Z.; Hesketh P. J.; Nair S. Wafer-Scale Process for Fabricating Arrays of Nanopore Devices. J. Micro/Nanolithogr., MEMS, MOEMS 2010, 9, 033011 10.1117/1.3486202.

[ref17] Zeng S.; Wen C.; Solomon P.; Zhang S.-L.; Zhang Z. Rectification of Protein Translocation in Truncated Pyramidal Nanopores. Nat. Nanotechnol. 2019, 14, 1056–1062. 10.1038/s41565-019-0549-0. 31591525

[ref18] Chang H.; Kosari F.; Andreadakis G.; Alam M. A.; Vasmatzis G.; Bashir R. DNA-Mediated Fluctuations in Ionic Current through Silicon Oxide Nanopore Channels. Nano Lett. 2004, 4, 1551–1556. 10.1021/nl049267c.

[ref19] Deng T.; Wang Y.; Chen Q.; Chen H.; Liu Z. Massive Fabrication of Silicon Nanopore Arrays with Tunable Shapes. Appl. Surf. Sci. 2016, 390, 681–688. 10.1016/j.apsusc.2016.07.171.

[ref20] Merchant C. A.; Healy K.; Wanunu M.; Ray V.; Peterman N.; Bartel J.; Fischbein M. D.; Venta K.; Luo Z.; Johnson A. T. C.; Drndić M. DNA Translocation through Graphene Nanopores. Nano Lett. 2010, 10, 2915–2921. 10.1021/nl101046t. 20698604

[ref21] Feng J.; Liu K.; Bulushev R. D.; Khlybov S.; Dumcenco D.; Kis A.; Radenovic A. Identification of Single Nucleotides in MoS_2_ Nanopores. Nat. Nanotechnol. 2015, 10, 1070–1076. 10.1038/nnano.2015.219. 26389660

[ref22] Bafna J. A.; Soni G. V.; Wanunu M. Fabrication of Low Noise Borosilicate Glass Nanopores for Single Molecule Sensing. PLoS ONE 2016, 11, e0157399 10.1371/journal.pone.0157399. 27285088 PMC4902259

[ref23] Arjmandi-Tash H.; Bellunato A.; Wen C.; Olsthoorn R.; Scheicher R.; Zhang S.-L.; Schneider G. F. Zero-Depth Interfacial Nanopore Capillaries. Adv. Mater. 2018, 30, 1703602 10.1002/adma.201703602. 29372574

[ref24] Choi J.; Lee C. C.; Park S. Scalable Fabrication of Sub-10 nm Polymer Nanopores for DNA Analysis. Microsyst. Nanoeng. 2019, 5, 12 10.1038/s41378-019-0050-9. 31057939 PMC6453903

[ref25] Plummer J. D.; Deal M. D.; Griffin P. B. Silicon VLSI Technology – Fundamentals, Practice and Modeling; Prentice Hall: Upper Saddle River, NJ, 2000.

[ref26] Habermehl S. Coefficient of Thermal Expansion and Biaxial Young’s Modulus in Si-Rich Silicon Nitride Thin Films. J. Vac. Sci. Technol., A 2018, 36, 021517 10.1116/1.5020432.

[ref27] Wang Y.; Tao J.; Tong S.; Sun T.; Zhang A.; Feng S. The Oxidation Kinetics of Thin Polycrystalline Silicon Films. J. Electrochem. Soc. 1991, 138, 214–219. 10.1149/1.2085542.

[ref28] Deal B. E.; Sklar M. Thermal Oxidation of Heavily Doped Silicon. J. Electrochem. Soc. 1965, 112, 430–435. 10.1149/1.2423562.

[ref29] Yuen C. Y.; Poon M. C.; Chan W. Y.; Qin M. Investigation of Grain Formation and Growth in Nickel-Induced Lateral Crystallization Process. J. Appl. Phys. 2002, 92, 6291 10.1063/1.1513881.

[ref30] Wang H.; Chan M.; Jagar S.; Poon V. M. C.; Qin M.; Wang Y.; Ko P. K. Super Thin-Film Transistor with SOI CMOS Performance Formed by a Novel Grain Enhancement Method. IEEE Trans. Electron Devices 2000, 47, 1580–1586. 10.1109/16.853034.

[ref31] Li S.; Zeng S.; Wen C.; Barbe L.; Tenje M.; Zhang Z.; Hjort K.; Zhang S.-L. Dynamics of DNA Clogging in Hafnium Oxide Nanopores. J. Phys. Chem. B 2020, 124 (51), 11573–11583. 10.1021/acs.jpcb.0c07756. 33315405 PMC7770817

[ref32] Kao D.-B.; McVittie J. P.; Nix W. D.; Saraswat K. C. Two-Dimensional Thermal Oxidation of Silicon. II. Modeling Stress Effects in Wet Oxides. IEEE Trans. Electron Devices 1988, 35, 25–37. 10.1109/16.2412.

[ref33] Deal B. E.; Grove A. S. General Relationship for the Thermal Oxidation of Silicon. J. Appl. Phys. 1965, 36, 3770–3778. 10.1063/1.1713945.

[ref34] Kao D.-B.; McVittie J. P.; Nix W. D.; Saraswat K. C. Two-Dimensional Thermal Oxidation of Silicon. I. Experiments. IEEE Trans. Electron Devices 1987, 34, 1008–1017. 10.1109/T-ED.1987.23037.

[ref35] O’Hanlon J. F. A User’s Guide to Vacuum Technology; 3rd ed.; John Wiley & Sons, Inc.: Hoboken, NJ, 2003.

[ref36] Biermann E.; Berger H. H.; Linke P.; Müller B. Oxide Growth Enhancement on Highly *n*-Type Doped Silicon under Steam Oxidation. J. Electrochem. Soc. 1996, 143, 1434–1442. 10.1149/1.1836656.

[ref37] Krzeminski C. D.; Han X.-L.; Larrieu G. Understanding of the Retarded Oxidation Effects in Silicon Nanostructures. Appl. Phys. Lett. 2012, 100, 263111 10.1063/1.4729410.

[ref38] Heidemeyer H.; Single C.; Zhou F.; Prins F. E.; Kern D. P.; Plies E. Self-Limiting and Pattern Dependent Oxidation of Silicon Dots Fabricated on Silicon-on-Insulator Material. J. Appl. Phys. 2000, 87, 4580–4585. 10.1063/1.373105.

[ref39] Cui H.; Wang C. X.; Yang G. W. Origin of Self-Limiting Oxidation of Si Nanowires. Nano Lett. 2008, 8, 2731–2737. 10.1021/nl8011853. 18680350

[ref40] Ho C. P.; Plummer J. D. Si/SiO_2_ Interface Oxidation Kinetics: A Physical Model for the Influence of High Substrate Doping Levels I. Theory. J. Electrochem. Soc. 1979, 126, 1516–1522. 10.1149/1.2129320.

[ref41] Mandurah M. M.; Saraswat K. C.; Helms C. R.; Kamins T. I. Dopant Segregation in Polycrystalline Silicon. J. Appl. Phys. 1980, 51, 5755–5763. 10.1063/1.327582.

[ref42] Kamgar A.; Baiocchi F. A.; Sheng T. T. Kinetics of Arsenic Activation and Clustering in High Dose Implanted Silicon. Appl. Phys. Lett. 1986, 48, 1090–1092. 10.1063/1.96607.

[ref43] Rabie M. A.; Haddara Y. M.; Carette J. A Kinetic Model for the Oxidation of Silicon Germanium Alloys. J. Appl. Phys. 2005, 98, 074904 10.1063/1.2060927.

[ref44] Kamohara S.; Kamigaki Y. Activation Energy Enhancement during Initial Silicon-Oxide Growth in Dry Oxygen. J. Appl. Phys. 1991, 69, 7871–7875. 10.1063/1.347520.

[ref45] Yao Y.; Wen C.; Pham N. H.; Zhang S.-L. On Induced Surface Charge in Solid-State Nanopores. Langmuir 2020, 36, 8874–8882. 10.1021/acs.langmuir.0c01189. 32646217

[ref46] Mao M.; Ghosal S.; Hu G. Hydrodynamic Flow in the Vicinity of a Nanopore Induced by an Applied Voltage. Nanotechnology 2013, 24, 245202 10.1088/0957-4484/24/24/245202. 23689946 PMC3738177

[ref47] Squires T. M.; Bazant M. Z. Induced-Charge Electro-Osmosis. J. Fluid Mech. 1999, 509, 217–252. 10.1017/S0022112004009309.

[ref48] Bazant M. Z.; Squires T. M. Induced-Charge Electrokinetic Phenomena: Theory and Microfluidic Applications. Phys. Rev. Lett. 2004, 92, 066101 10.1103/PhysRevLett.92.066101. 14995255

[ref49] Wen C.; Zeng S.; Arstila K.; Sajavaara T.; Zhu Y.; Zhang Z.; Zhang S.-L. Generalized Noise Study of Solid-State Nanopores at Low Frequencies. ACS Sens. 2017, 2, 300–307. 10.1021/acssensors.6b00826. 28723146

[ref50] Wen C.; Zhang Z.; Zhang S.-L. Physical Model for Rapid and Accurate Determination of Nanopore Size *via* Conductance Measurement. ACS Sens. 2017, 2, 1523–1530. 10.1021/acssensors.7b00576. 28974095

[ref51] Pabit S. A.; Qiu X.; Lamb J. S.; Li L.; Meisburger S. P.; Pollack L. Both Helix Topology and Counterion Distribution Contribute to the More Effective Charge Screening in DsRNA Compared with DsDNA. Nucleic Acids Res. 2009, 37, 3887–3896. 10.1093/nar/gkp257. 19395592 PMC2709557

[ref52] Di Fiori N.; Squires A.; Bar D.; Gilboa T.; Moustakas T. D.; Meller A. Optoelectronic Control of Surface Charge and Translocation Dynamics in Solid-State Nanopores. Nat. Nanotechnol. 2013, 8, 946–951. 10.1038/nnano.2013.221. 24185943 PMC3998374

[ref53] Carlsen A. T.; Zahid O. K.; Ruzicka J.; Taylor E. W.; Hall A. R. Interpreting the Conductance Blockades of DNA Translocations through Solid-State Nanopores. ACS Nano 2014, 8, 4754–4760. 10.1021/nn501694n. 24758739

[ref54] Larkin J.; Henley R. Y.; Muthukumar M.; Rosenstein J. K.; Wanunu M. High-Bandwidth Protein Analysis Using Solid-State Nanopores. Biophys. J. 2014, 106, 696–704. 10.1016/j.bpj.2013.12.025. 24507610 PMC3944622

[ref55] Wanunu M.; Morrison W.; Rabin Y.; Grosberg A. Y.; Meller A. Electrostatic Focusing of Unlabelled DNA into Nanoscale Pores Using a Salt Gradient. Nat. Nanotechnol. 2010, 5, 160–165. 10.1038/nnano.2009.379. 20023645 PMC2849735

[ref56] Bell N. A. W.; Chen K.; Ghosal S.; Ricci M.; Keyser U. F. Asymmetric Dynamics of DNA Entering and Exiting a Strongly Confining Nanopore. Nat. Commun. 2017, 8, 380 10.1038/s41467-017-00423-9. 28855527 PMC5577289

