# Supplementary material for: Self-Limited Formation of Bowl-Shaped Nanopores for Directional DNA Translocation
Source: ACS Nano. 2021 Nov 11;15(11):17938–46. doi: 10.1021/acsnano.1c06321 (PMC8613906; doi:10.1021/acsnano.1c06321)
Supplement: Supplementary file 1 — nn1c06321_si_001.pdf [file nn1c06321_si_001.pdf]

Supporting Information

Self-Limited Formation of Bowl-Shaped Nanopores for Directional DNA Translocation

*Ngan Hoang Pham[1,3], Yao Yao[1,3], Chenyu Wen[1,3], Shiyu Li[1], Shuangshuang Zeng[1], Tomas Nyberg[1], Tuan Thien Tran[2], Daniel Primetzhofer[2], Zhen Zhang[1] & Shi-Li Zhang[1]**

[1]Division of Solid-State Electronics, Department of Electrical Engineering, Uppsala University, SE-751 03 Uppsala, Sweden

[2]Division of Applied Nuclear Physics, Department of Physics and Astronomy, Uppsala University, SE-751 20 Uppsala, Sweden

[3]These authors contributed equally: Ngan Hoang Pham, Yao Yao, Chenyu Wen.

* To whom correspondence should be addressed: shili.zhang@angstrom.uu.se.

Table of Contents:

Supporting Note S1. Resistance model for BNP pore size and surface charge extraction.

Supporting Note S2. Dominance of electrophoretic force during dsDNA translocation.

Supporting Note S3 Estimation of capture radius of nanopore for DNA translocation

Figure S1 Ionic rectification ratio of fabricated BNPs with different diameters.

Figure S2 Electric field distribution in a 5 nm-diameter BNP.

Figure S3. Noise power spectrum density (PSD) for a fabricated 4.4 nm BNP.

Figure S4. Calculation of the effective transport length of the BNPs.

Figure S5. Current-voltage characteristic curves of the 4.4 nm BNP and conductance-conductivity curve.

Figure S6. Distribution of the total force acting on a base pair of ds-DNA in a BNP of d_p=3 nm in 500 mM KCl.

Figure S7. Distribution of the electrophoretic force and the electroosmotic force along the central axis of the BNP of d_p=3 nm at V=+500 mV.

Figure S8. Comparison between the driven forces in BNP and TCP at different bias voltages.

Figure S9. Scatter plots of amplitude of translocation spikes of λ-DNA translocating an 8 nm BNP.

Figure S10 Capture surface of dsDNA translocation in an 8 nm BNP.

Figure S11. Translocation traces and typical waveform of λ-DNA translocating the 4.4 nm BNP.

Supporting Figure S1

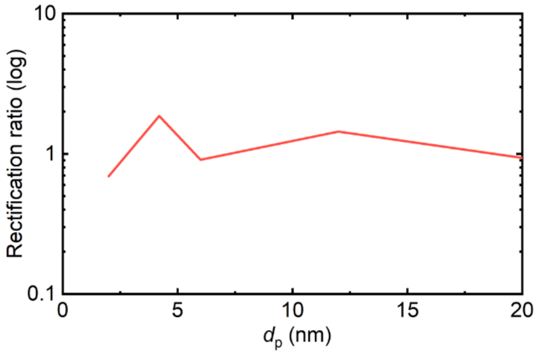

Figure S1. Ionic rectification ratio of fabricated BNPs of d_p=2-20 nm in electrolyte of KCl concentration of 500 mM. The rectification ratio is defined as $R_{rec}=|I_{0.5V}/I_{-0.5V}|$.

Supporting Figure S2

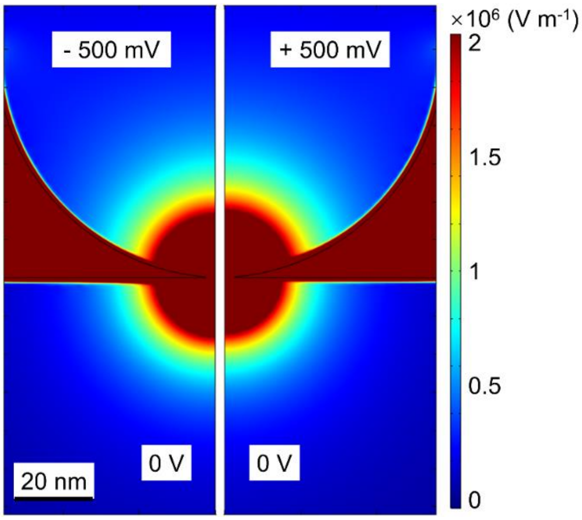

Figure S2. Electric field distribution in a 5 nm-diameter BNP at +500 mV and -500 mV bias voltage.

Supporting Figure S3

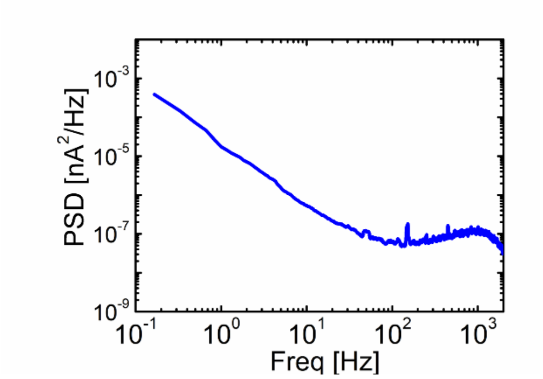

Figure S3. Noise power spectrum density (PSD) for a fabricated 4.4 nm BNP in 500 mM KCl solution under 100 mV bias. Similar noise characteristics can be found in our previous noise study of SiN_x nanopores[2].

Supporting Note S1. Resistance model for extraction of the BNP size and surface charge

Following the concept of our previous publication[1], the keystone to establish the resistance model for the BNPs is to calculate the effective transport length, L_{eff}. The L_{eff} of a nanopore is defined as the distance between the two points along the central axis of the nanopore where the electric field intensity is e^{-1} of its maximum value.

As there is neither source nor sink along the path of electric field lines apart from the surface charge, the integration of electric flied flux on any equi-electric-field-intensity-surface should be the same. Thus, the task to find the position of an equi-electric-field-intensity-surface where the electric field is e^{-1} of its maximum can be converted to finding an equi-electric-field-intensity-surface whose area is e times the area of the smallest restriction of the nanopore.

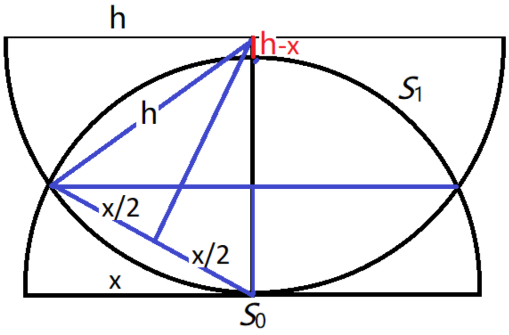

Figure S4. Calculation of L_{eff} of the BNPs.

As shown in Fig. S4, the equi-electric-field-intensity-surface in a bowl can be approximated as the hemisphere interception with the hemisphere of the bowl. Assume that the centre of the equi-electric-field-intensity hemisphere is located at the bottom centre of the bowl. From the geometrical relationship, the area of the intercepted equi-electric-field-intensity hemisphere can be expressed as

$$S_1 = 2\pi x^2 (1 - \frac{x}{2h}) \tag{S1}$$

Thus,

$$\frac{S_1 + S_0}{S_0} = e \tag{S2}$$

with

$$S_0 = \frac{\pi d_p^2}{4} \tag{S3}$$

as the area of the smallest restriction of the BNP where the electric field reaches its maximum. d_p is the diameter of this smallest constriction in circular shape.

x can be found by substituting Eqs. S1 and S2 into Eq. S3. One of the real roots of this third-order equation is the desired value. By considering the electric field below the smallest restriction, the total L_{eff} becomes:

$$L_{eff} = x + 0.46 d_p \tag{S4}$$

Hence, the resistance of the BNP is

$$R = \frac{4\rho L_{eff}}{\pi d_p^2} \tag{S5}$$

where, ρ is the resistivity of the electrolyte.

Extraction of surface charge density from conductance measurement

According to the literature, the conductance of a nanopore can be contributed by two parts: bulk conductance G_b and surface conductance G_s as[2–4]:

$$G = G_b + G_s \tag{S6}$$

wherein, $G_b=1/R$ while G_s is determined by the surface charge density and corresponding ion mobility[3,4]:

$$G_s = \mu\sigma \frac{\pi d_p}{h} \tag{S7}$$

where, σ is the surface charge density and μ the mobility of ions in the diffuse layer of EDL. However, the BNP has an uneven cross-sectional area. L_{eff} is used instead of h in Eq. S7 for estimation:

$$G_s = \mu\sigma \frac{4a}{L_{eff}} \; (h > L_{eff}) \tag{S8}$$

By fitting the conductance G *vs.* conductivity $1/\rho$ data with Eq. S8, d_p and σ can be extracted.

Supporting Figure S5

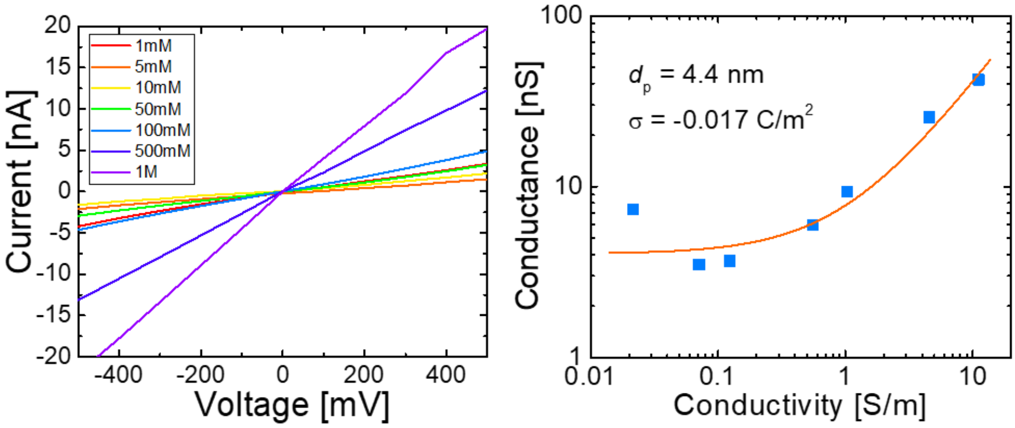

Figure S5. Current-voltage (*I*-*V*) characteristic curves of the 4.4 nm BNP in electrolytes of different KCl concentrations (left). Conductance of the 4.4 nm BNP *versus* conductivity of the electrolytes from which d_p and σ were extracted with the assistance of the nanopore resistance model (right).

Supporting Note S2. Dominance of electrophoretic force during dsDNA translocation

Dominant factor

Electrophoretic force:

$$F_{el} = qE_z \tag{S9}$$

where, q is the charge of the translocating object and E_z the electric field intensity along the vertical direction, *i.e.* z-direction.

Electroosmotic force:

$$F_{eof} = A\eta \frac{\partial w}{\partial n} \tag{S10}$$

where, A is the surface area of the object, η the viscosity of water, w the vertical component of the water velocity and n the normal of the translocating object surface. Consider one base pair of a double-stranded DNA (ds-DNA), its side area A that interacts with EOF is:

$$A = 2\pi r d_s \tag{S11}$$

where, r=1.1 nm is the radius of DNA and d_s=0.33 nm the length of one DNA base pair. The velocity gradient of EOF can be approximated by w_{max}/λ_D where w_{max} is the maximum vertical component of the water velocity and λ_D the Debye length. In 500 mM KCl, λ_D=0.4 nm.

The effective charge of ds-DNA is reduced from -2e/bp to -0.49e/bp by considering the screening effect from the electrolyte. Thus, the total force acting on one base pair of ds-DNA is:

$$F = F_{el} + F_{eof} \tag{S12}$$

For a 3 nm-diameter BNP, the distribution of total force on such a base pair is shown in Fig. S6. The movement of DNA is governed by the electrophoretic force due to its high charge density. For larger BNPs, the EOF effect is weaker while the electrophoretic force stays unchanged. Thus, the DNA is always driven by the electrophoretic force.

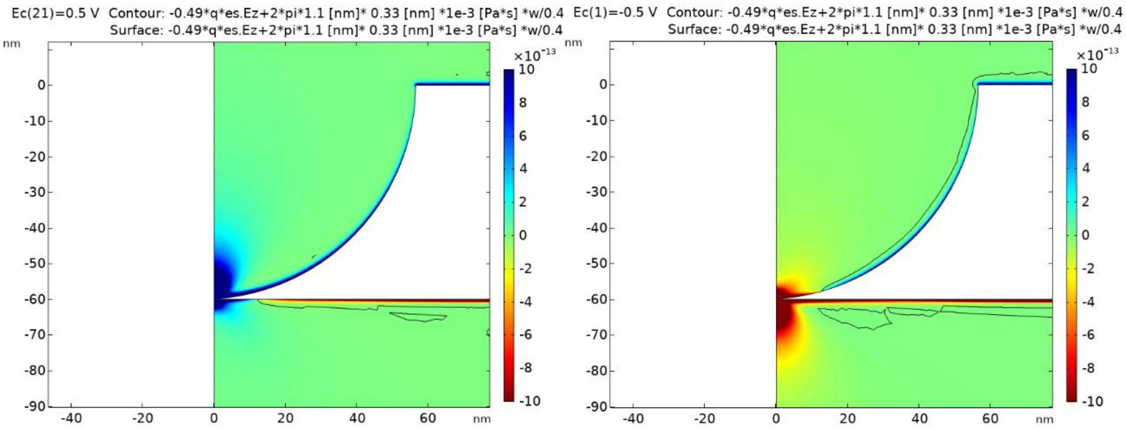

Figure S6. Distribution of the total force F=F_{el}+F_{eof} acting on a base pair of ds-DNA at (left) V=+500 mV and (right) V=-500 mV in a BNP of d_p=3 nm in 500 mM KCl.

Competition between electrophoretic force and electroosmotic force

In the case with a BNP of d_p=3 nm, the maximum value of the electric field is 7×10^7 V/m. Thus, the maximum electrophoretic force is $F_{el}=0.49\times1.6\times10^{-19}\times7\times10^7=5.5$ pN.

The maximum water flow rate is 0.35 m/s, and the maximum electroosmotic force is F_{eof} =1.9 pN according to Eq. S10, which is 35% of F_{el}. It is worth noting that F_{eof} of the BNP is almost 10 times larger than that in a counterpart TCP also of d_p=3 nm, because the EOF rate is of the order of 0.01 m/s.

For a better comparison, the F_{el} and F_{eof} on a base pair of ds-DNA is plotted along the central axis of the BNP of d_p=3 nm, as shown in Fig. S7. Although the electrophoretic force governs the DNA translocation, the electroosmotic force is of the same order of magnitude and is anticipated to play a significant role in modulating the movement of DNA.

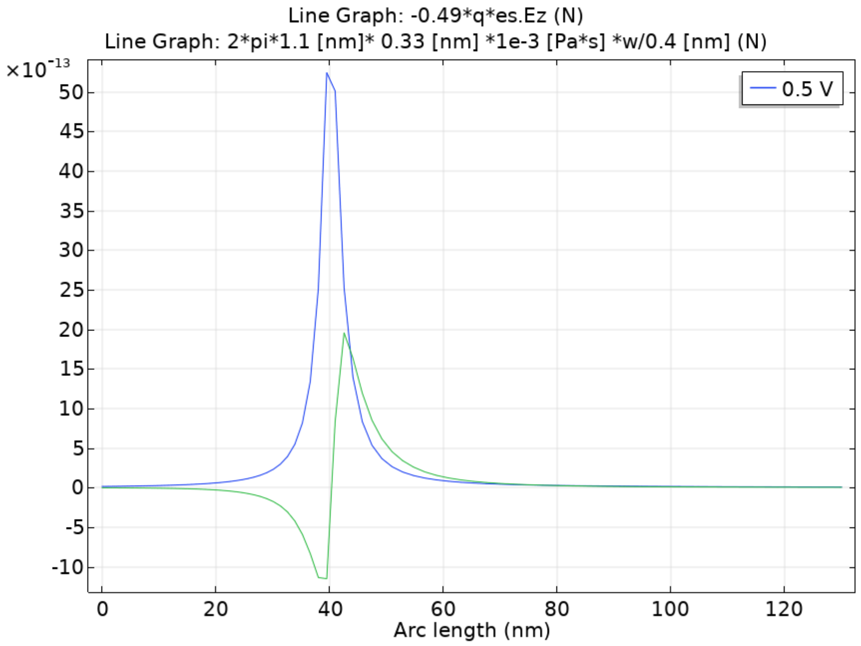

Figure S7. Distribution of the electrophoretic force (blue line) and the electroosmotic force (green line) along the central axis of the BNP of d_p=3 nm at V=+500 mV. The smallest restriction of the BNP is seen to locate at the position of 40 nm and the large opening is at the position of 100 nm.

Supporting Figure S8

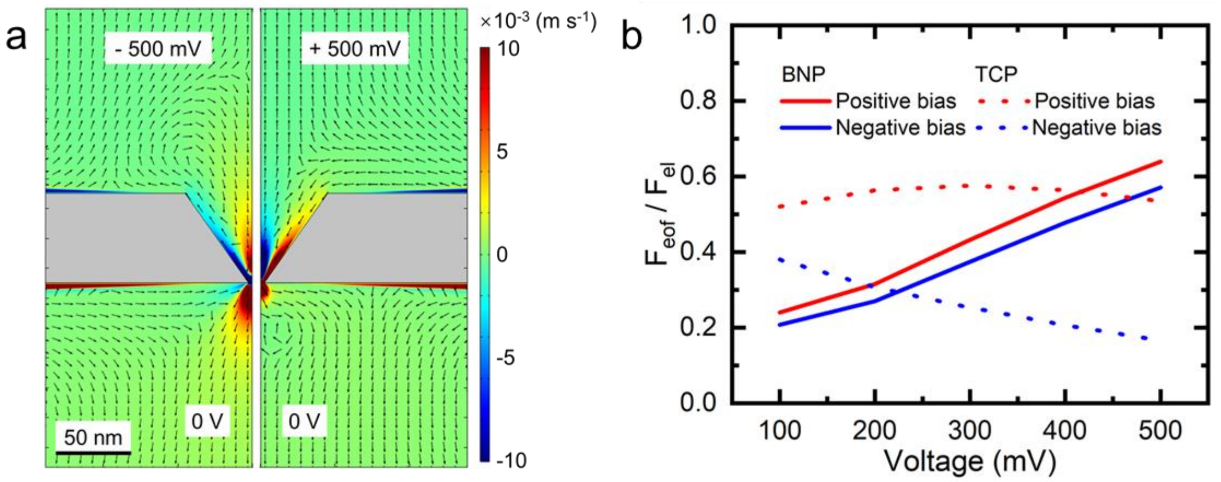

Figure S8. (a) Electroosmotic flow velocity distribution at V=−500 mV (left part on the left) and V=+500 mV (right part on the left) of a truncated conical nanopore (TCP) of d_p=5 nm. (b) Comparison between BNP and TCP for dependence on bias voltage of the maximum ratio of the electroosmotic force to the opposite electrophoretic force at the same position.

For the TCP in Fig. S8a, the electroosmotic vortexes are found neither in the lower reservoir near the smallest restriction of the nanopore at positive bias nor inside the nanopore above the smallest restriction at negative bias. Clearly different from BNPs, the EOF along the surface of the sidewall can spread to and fill up the smallest restriction of the TCP for both bias conditions and thereby contribute to the electroosmotic force that opposes the DNA translocation. However, a stream of water flow retarding the DNA translocation appears along the central axis of the BNP above and below the smallest restriction at negative and positive bias, respectively. For a better comparison between the driving forces in BNP and TCP, the maximum ratio of electroosmotic force to electrophoretic force along the central axes of the nanopores is shown in Fig. S8b at different bias voltages. The difference in this ratio at positive and negative biases is much larger for the TCP than for the BNP. For the TCP at positive bias seen in Fig. S8a, the EOF generated along the sidewall is seamlessly connected to the lower reservoir without any counter flow. This smooth connection leads to a higher velocity of EOF and correspondingly a stronger electroosmotic force than those in the counterpart BNP. Furthermore, higher biases result in larger maximum ratios for the BNP, which is significantly different from the TCP.

Supporting Figure S9

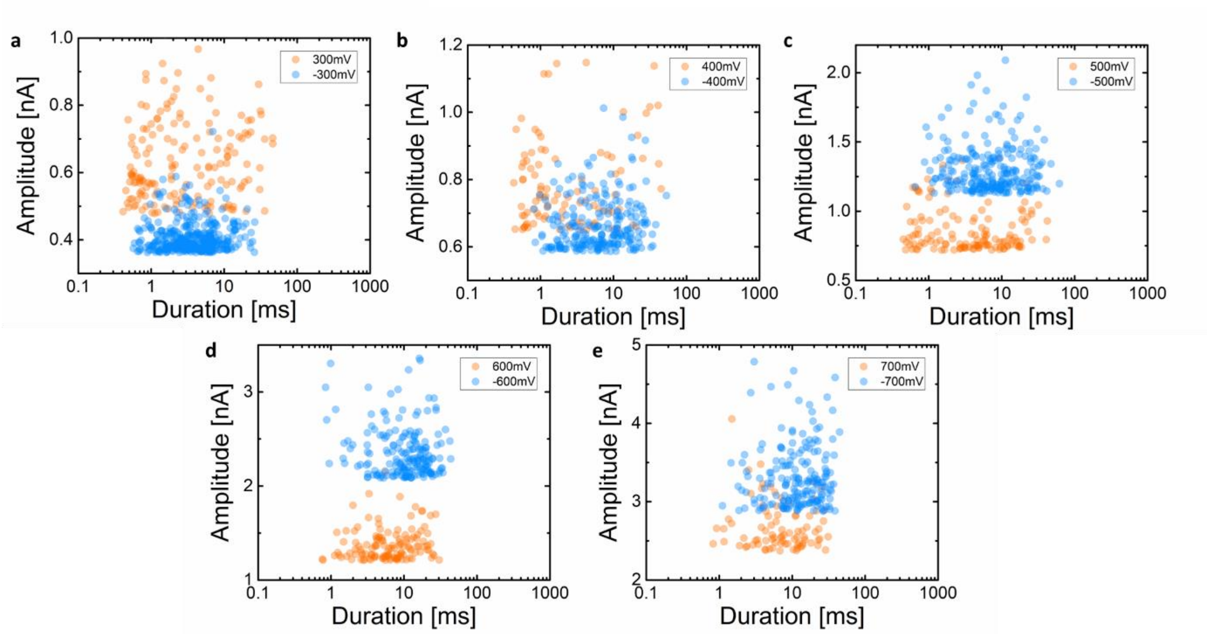

Figure S9. Scatter plots of amplitude of translocation spikes of λ-DNA translocating an 8 nm BNP under bias of (a) ±300 mV; (b) ±400 mV; (c) ±500 mV; (d) ±600 mV; (e) ±700 mV.

Supporting Note S3. Estimation of capture radius of nanopore for DNA translocation

The effective capture radius (r^*) of the analyte translocation can be defined as[7]

$$r^* = \frac{d_p^2 \mu}{8hD} \Delta V \qquad \text{(S13)}$$

where, d_p is the pore diameter, h the thickness of the pore and ΔV the voltage applied to the electrodes, μ the DNA electrophoretic mobility, and D the DNA diffusion coefficient. D/μ is the potential where the capture hemisphere locates, *i.e.*, $V(r^*) = D/\mu$. Considering a segment of dsDNA with its persistence length, 35 nm,[6] the position of the capture hemisphere should locate at the position where the energy of dsDNA segment gained from the electric field is equal to the thermal energy kT.[8]

$$snqV(r^*) = kT \qquad \text{(S14)}$$

where, n is the number of unit charges on the DNA segment, s the ratio of effective charge after the ion screening effect, and q the unit charge. For a segment of 35 nm dsDNA, $n = 35/0.34\times2 \approx 200$. $s = 1/4.1 = 0.25$.[9]

In this way, $V(r^*) = D/\mu = 5.4\times10^{-4}$ V. For the λ-DNA translocation through an 8 nm BNP that only the thickness near the orifice is taken into consideration, the radius of capture hemisphere is around 1.2 μm, *i.e.*, 120 nm at 100 mV and 0.6 μm at 500 mV. In the simulation, we can directly measure the radius of capture hemisphere, which is just the radius of equipotential surface of $D/\mu = 5.4\times10^{-4}$ V. As shown in Fig. S10, the capture radius r^* is around 0.7 μm at -500 mV and 0.6 μm at +500 mV, which coincides with the theoretical estimation very well. It also indicates higher capture rate (translocation frequency) at negative bias.

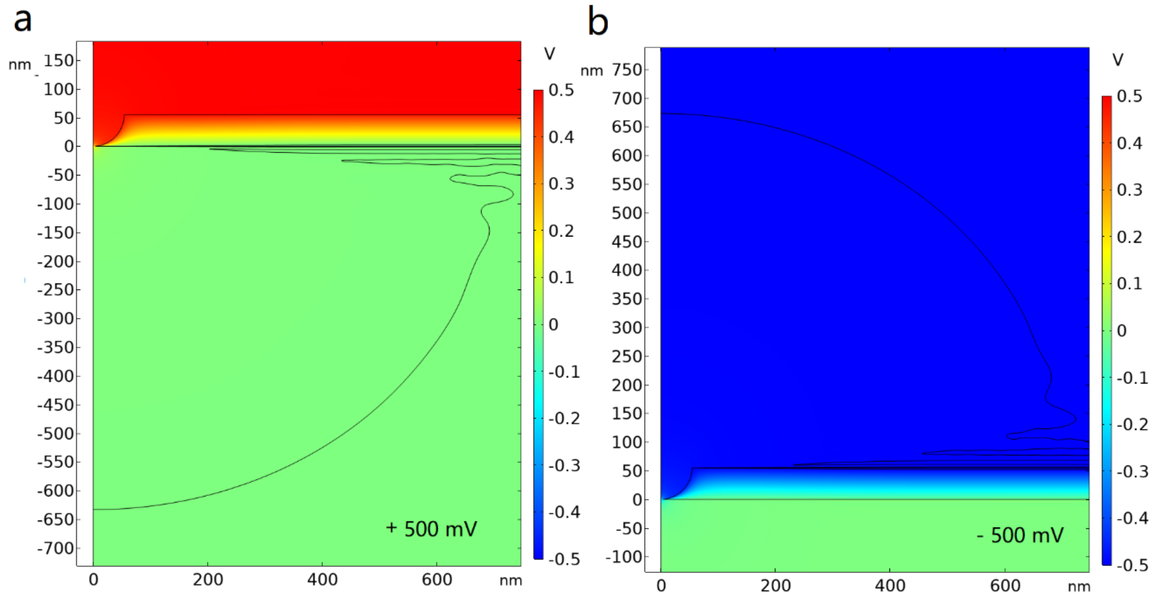

Figure S10. Electrical potential distribution in an 8 nm-diameter BNP at (a) +500 mV and (b) -500 mV bais. The black line in corresponding figure marks the equipotential surface of $D/\mu = 5.4\times10^{-4}$ V, which can be approximated as the capture surface for dsDNA.

Supporting Figure S11

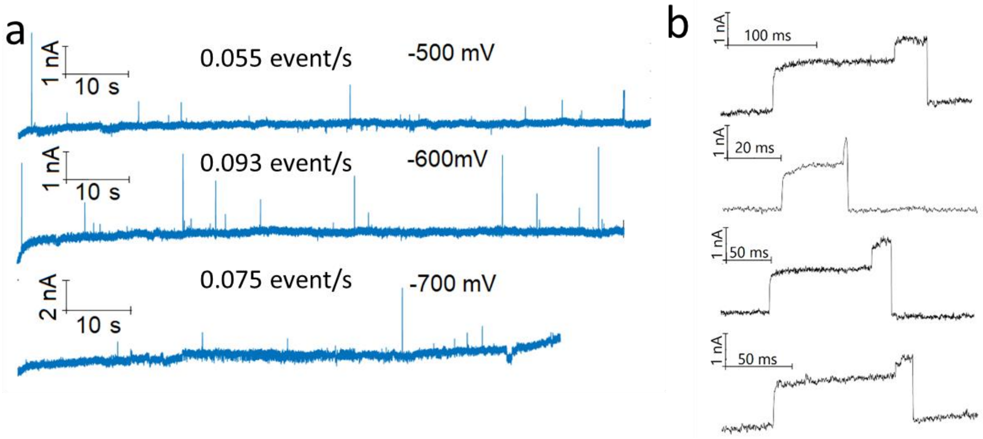

Figure S11. Translocation traces and typical waveform of λ-DNA translocating the 4.4 nm BNP. (a) Current traces showing spikes generated by the translocation at different negative biases. (b) Examples of the frequently observed translocation waveforms typically showing a long low-blockage period followed by a short high-blockage pulse that falls immediately back to the baseline.

Translocation experiments were first carried out using the d_p=4.4 nm BNP (Fig. S11a) with λ-DNA. Typical translocation spikes in Fig. S11a could be observed only at negative biases, which can be accounted for by invoking the 4-fold difference in the ratio of total force at negative bias to that at positive bias (Fig. 4c in the main text). Frequently observed translocation waveforms displayed in Fig. S11b are characterized by a long low-level blockage followed by a short high-level one falling directly back to the baseline. The 16-μm-long λ-DNA with 48,502 base pairs soaked in electrolytes can convolute into a loose clump of 1.2 μm in gyration diameter.[5] With a persistence length of 35 nm typical for double-stranded DNA,[6] part of the λ-DNA can dwell in the bowl and occupies a considerable volume in the high-electric-field region of the BNP. This behavior can render a significant semi-blockade responsible for the long low-level blockage in Fig. S11b. Although the 35-nm persistence length makes bending of the λ-DNA unlikely to fit into the 4.4 nm pore, the λ-DNA may slightly move and rotate until eventually threading through the BNP and causing the short high-level blockage. Lacking such a dynamic mechanism in the absence of a similar bowl below the BNP can explain no observed translocation from the lower reservoir, at positive bias.

Supporting references

(1) Wen, C.; Zhang, Z.; Zhang, S.-L. Physical Model for Rapid and Accurate Determination of Nanopore Size *via* Conductance Measurement. *ACS Sens.* **2017**, *2*, 1523–1530.

(2) Wen, C.; Zeng, S.; Arstila, K.; Sajavaara, T.; Zhu, Y.; Zhang, Z.; Zhang, S.-L. Generalized Noise Study of Solid-State Nanopores at Low Frequencies. *ACS Sens.* **2017**, *2*, 300–307.

(3) Smeets, R. M. M.; Keyser, U. F.; Krapf, D.; Wu, M.-Y.; Dekker, N. H.; Dekker, C. Salt Dependence of Ion Transport and DNA Translocation through Solid-State Nanopores. *Nano Lett.* **2006**, *6*, 89–95.

(4) Ivanov, A. P.; Instuli, E.; McGilvery, C. M.; Baldwin, G.; McComb, D. W.; Albrecht, T.; Edel, J. B. DNA Tunneling Detector Embedded in a Nanopore. *Nano Lett.* **2011**, *11*, 279–285.

(5) Tree, D. R.; Muralidhar, A.; Doyle, P. S.; Dorfman, K. D. Is DNA a Good Model Polymer? *Macromolecules* **2013**, *46*, 8369–8382.

(6) Guilbaud, S.; Salomé, L.; Destainville, N.; Manghi, M.; Tardin, C. Dependence of DNA Persistence Length on Ionic Strength and Ion Type. *Phys. Rev. Lett.* **2019**, *122*, 028102.

(7) Wanunu, M.; Morrison, W.; Rabin, Y.; Grosberg, A. Y.; Meller, A. Electrostatic Focusing of Unlabelled DNA into Nanoscale Pores Using a Salt Gradient. *Nat. Nanotechnol.* **2010**, *5*, 160–165.

(8) Grosberg, A. Y.; Rabin, Yitzhak. DNA Capture into a Nanopore: Interplay of Diffusion and Electrohydrodynamics. *J. Chem. Phys.* **2010**, *133*, 165102.

(9) Wen, C.; Zhang, S.-L. Fundamentals and Potentials of Solid-State Nanopores: A Review. *J. Phys. D: Appl. Phys.* **2021**, *54*, 023001
